# Acidified Nitrite Contributes to the Antitumor Effect of Cold Atmospheric Plasma on Melanoma Cells

**DOI:** 10.3390/ijms22073757

**Published:** 2021-04-04

**Authors:** Tom Zimmermann, Lisa A. Gebhardt, Lucas Kreiss, Christin Schneider, Stephanie Arndt, Sigrid Karrer, Oliver Friedrich, Michael J. M. Fischer, Anja-Katrin Bosserhoff

**Affiliations:** 1 Emil-Fischer-Center, Institute of Biochemistry, University of Erlangen-Nuernberg, 91054 Erlangen, Germany; tom.zimmermann@fau.de (T.Z.); schneiderchristin@gmx.de (C.S.); 2 Institute of Physiology and Pathophysiology, University of Erlangen-Nuernberg, 91054 Erlangen, Germany; lisa.a.gebhardt@fau.de (L.A.G.); michael.jm.fischer@meduniwien.ac.at (M.J.M.F.); 3 Department of Medicine I, University Clinics Erlangen, 91054 Erlangen, Germany; lucas.kreiss@fau.de; 4 Institute of Medical Biotechnology, University of Erlangen-Nuernberg, 91052 Erlangen, Germany; oliver.friedrich@mbt.uni-erlangen.de; 5 Department of Dermatology, University Hospital of Regensburg, 93053 Regensburg, Germany; stephanie.arndt@ukr.de (S.A.); sigrid.karrer@ukr.de (S.K.); 6 Institute of Physiology, Medical University of Vienna, 1090 Vienna, Austria; 7 Comprehensive Cancer Center (CCC) Erlangen-EMN, 91054 Erlangen, Germany

**Keywords:** cold atmospheric plasma, malignant melanoma, acidification, nitrite, acidified nitrite, nitration, membrane damage

## Abstract

Cold atmospheric plasma (CAP) is partially ionized gas near room temperature with previously reported antitumor effects. Despite extensive research and growing interest in this technology, active components and molecular mechanisms of CAP are not fully understood to date. We used Raman spectroscopy and colorimetric assays to determine elevated nitrite and nitrate levels after treatment with a MiniFlatPlaster CAP device. Previously, we demonstrated CAP-induced acidification. Cellular effects of nitrite and strong extracellular acidification were assessed using live-cell imaging of intracellular Ca^2+^ levels, cell viability analysis as well as quantification of p21 and DNA damage. We further characterized these observations by analyzing established molecular effects of CAP treatment. A synergistic effect of nitrite and acidification was found, leading to strong cytotoxicity in melanoma cells. Interestingly, protein nitration and membrane damage were absent after treatment with acidified nitrite, thereby challenging their contribution to CAP-induced cytotoxicity. Further, phosphorylation of ERK1/2 was increased after treatment with both acidified nitrite and indirect CAP. This study characterizes the impact of acidified nitrite on melanoma cells and supports the importance of RNS during CAP treatment. Further, it defines and evaluates important molecular mechanisms that are involved in the cancer cell response to CAP.

## 1. Introduction

Cold atmospheric plasma (CAP) consists of a heterogeneous mixture of reactive oxygen (ROS) and nitrogen (RNS) species, as well as other ions, uncharged particles, and small amounts of radiation in ultraviolet and infrared ranges. Manifold effects of CAP have been described in the past, mainly referring to strong antibacterial, antiviral, and antifungal action [1,2,3]. Significant efforts have been made to evaluate and use its positive impact on wound healing [4,5,6], dental health [7,8], and regenerative medicine [9]. Furthermore, beneficial effects of plasma treatment have been reported both in vitro and in vivo for numerous cancer types, including malignant melanoma [10,11,12], colon [13,14], and brain tumors [15,16,17]. Given the constantly high incidence and mortality of such malignancies, and their enormous burden on the patient as well as the health care system, CAP technology displays a promising approach to the development of novel therapeutic treatments. The potential impact of an oncological application is highlighted by recent studies showing that chemo-resistance might be challenged directly via apoptosis [18,19] and indirectly by restoration of chemo-sensitivity [20]. Furthermore, it was reported that CAP shows strong selectivity against cancer cells, while healthy cells remain largely unaffected [21,22,23]. Such preferential killing of tumor cells, however, remains controversial as treatment conditions and cell culture media largely affect the potency of CAP [24]. Despite extensive research and ongoing advances in plasma medicine, the exact molecular mechanisms of CAP treatment are still unknown. In terms of malignant melanoma, multiple studies have contributed to a better understanding of CAP effects and underlying molecular mechanisms. For example, it was shown that melanoma cells enter apoptosis in response to DNA damage and mitochondrial dysfunction caused by CAP-induced ROS and RNS [25,26]. Our group previously reported dose-dependent effects of CAP ranging from senescence to apoptosis [11]. On a molecular level, the establishment of cellular senescence was tightly linked to an immediate elevation of cytoplasmic Ca^2+^ levels, mainly originating from intracellular stores [27]. In comparison to direct treatment of melanoma cells, indirect treatment by application of CAP-treated physiological buffers showed similar but attenuated results, indicating that many of the activating agents are able to dilute in aqueous solutions and remain fairly stable. This was supported by the finding that such CAP-treated buffers do not lose their biological effect if their application is delayed for one hour. Another study revealed strong extracellular acidification during CAP treatment to be essential for its effect on melanoma cells [28]. However, since the exact molecular and cellular mechanisms are still unknown, further research is required to enable the development of plasma-based tumor therapy and valid plasma devices for such approaches. The aim of this study was to characterize reactive species involved in the CAP effect on melanoma cells and to further understand the CAP-induced mechanisms as a basis for the generation of personalized plasma therapy.

## 2. Results

### 2.1. CAP Induces Production of Nitrate and Nitrite in Aqueous Solutions

**Figure 1:**
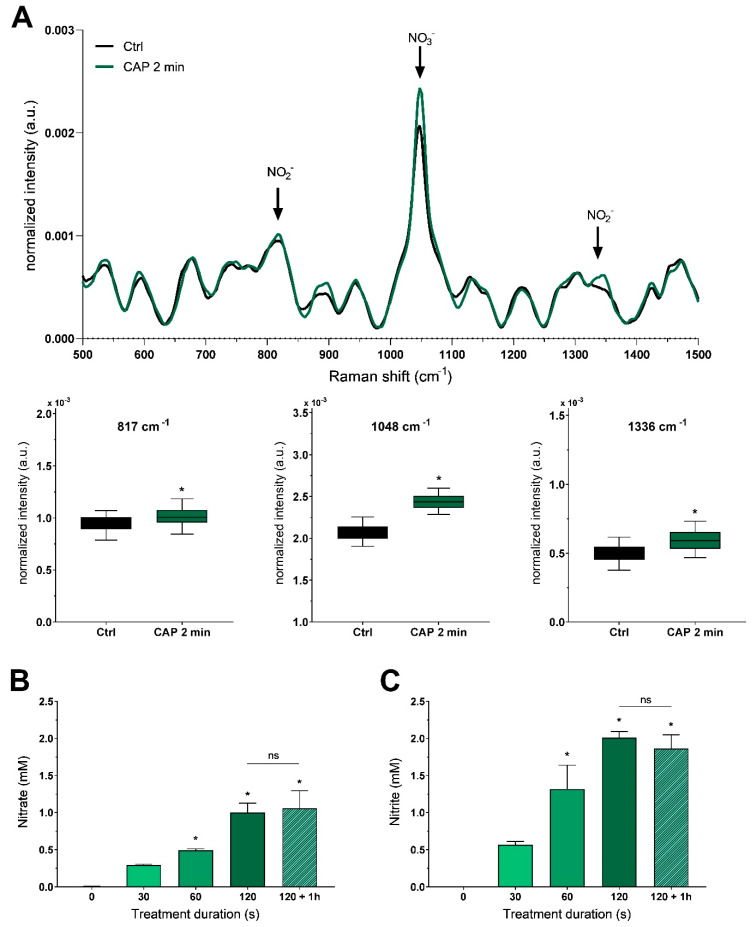
CAP treatment causes the production of nitrate and nitrite. (**A**) Raman spectroscopy of untreated extracellular solution (Ctrl) versus a solution treated with 2 min CAP. Traces are mean only, and box plots are mean with 95% confidence interval (Student’s *t*-test, *n* = 300). (**B**) Photometric quantification of nitrate and (**C**) nitrite after different doses of CAP. Stability of these molecules was assessed 1 h after treatment (F_(4,10)_ = 29.31 and F_(4,10)_ = 29.63, both *p* < 0.0001). Bars are shown as mean ± SEM (ANOVA followed by Tukey’s HSD post-hoc test vs. no treatment, *n* = 3, *: *p* <0.05, ns: not significant (*p* > 0.05)).

Recently, we described the effects of surface micro discharge (SMD) generated CAP on tumor cells, linking these to the induction of reactive species. For an unbiased characterization of CAP-induced production of ROS and RNS, we used Raman spectroscopy. Even though this approach was not able to detect reactive species with a short lifetime, significantly elevated levels of nitrate (1048 cm^−1^) and nitrite (817 cm^−1^ and 1336 cm^−1^) were found after 2 min CAP treatment (Figure 1A). The validity of these findings was confirmed using standard solutions of potassium nitrate and sodium nitrite (Figures S1 and S2). An established colorimetric assay based on the transnitration of salicylic acid [29] was then used to validate and quantify the observed nitrate production. We determined a dose-dependent increase in nitrate levels reaching approximately 1 mM nitrate after 2 min CAP treatment (Figure 1B). Nitrite levels were assessed using a colorimetric assay based on the Griess diazotization reaction. A similar dose-dependency was observed, resulting in nitrite levels of 2 mM after 2 min CAP (Figure 1C). In previous studies, we could show that CAP-treated solutions retained a substantial fraction of their cellular effects even 1 h after incubation [27]. We, therefore, assessed the stability of nitrate and nitrite and found high stability of the CAP-induced substances (Figure 1B,C).

### 2.2. Nitrite and Acidification Have Synergistic Effects on Ca^2+^ Influx and Cytotoxicity

**Figure 2:**
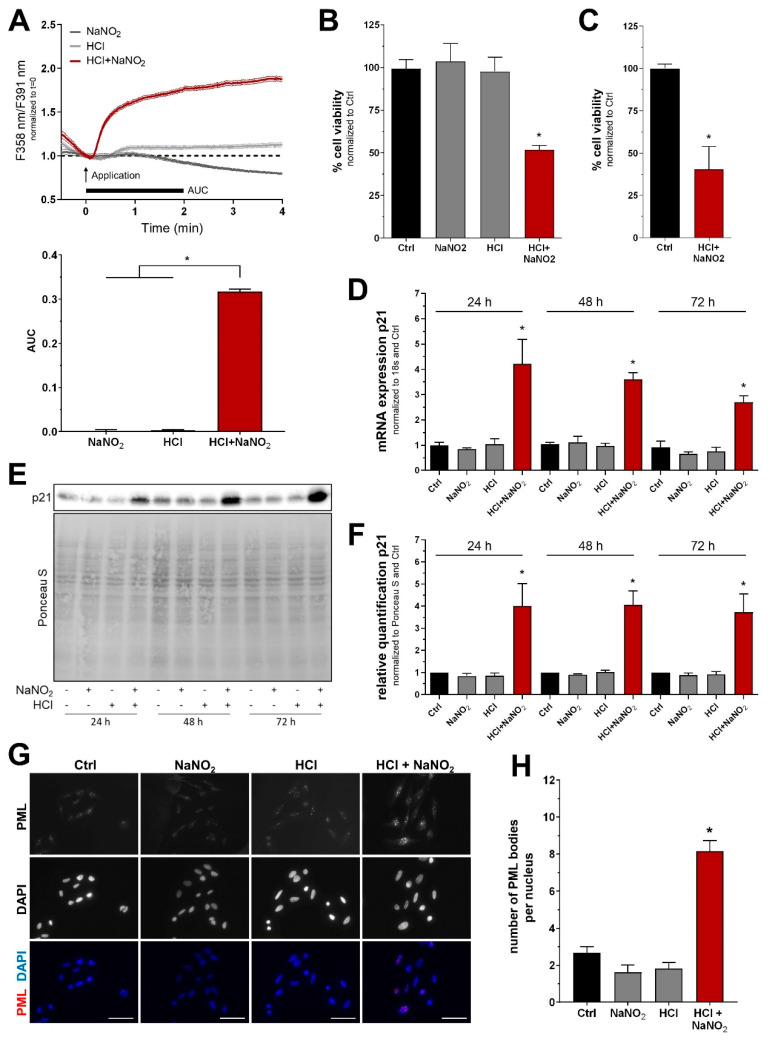
Nitrite and acidification have synergistic effects on melanoma cells. (**A**) Time course of cytoplasmic Ca^2+^ levels due to a 4-min treatment with acidic ECS (HCl), nitrite solution (NaNO_2_), or a combination of both. Ca^2+^ levels were quantified by calculating the area under the curve (AUC) of the first 120 s after the start of the application (F_(2,828)_ = 1891, *p* <0.0001, *n* = 260–293). (**B**) Cell viability analysis 24 h after a 5 min treatment with untreated ECS (Ctrl) or solutions described in (**A**) (F_(3,8)_ = 10.93, *p* = 0.0033). (**C**) Cell viability analysis 24 h after a 5 min treatment with phosphate-buffered ECS without HEPES (Ctrl) or a combination of acidic phosphate-buffered ECS without HEPES and nitrite (Student’s *t*-test). (**D**) Expression analysis of p21 during the time span of 24–72 h after treatment (F_(11,24)_ = 13.82, *p* < 0.0001). (**E**,**F**) Western blot analysis and quantification of p21 protein levels with similar incubation time as (**D**) (F_(11,24)_ = 10.44, *p* < 0.0001). (**G**,**H**) Immunofluorescent stainings of PML and DAPI to assess DNA damage. The amount of PML nuclear bodies was quantified in the bar chart (F_(3,8)_ = 55.13, *p* < 0.0001). Scale bars: 50 µm. Traces are mean with 95% confidence interval, bars are mean ± SEM (ANOVA followed by Tukey’s HSD post-hoc test vs. Ctrl, *n* = 3, *: *p* < 0.05).

Next, we assessed the effects of nitrate and nitrite on the cytoplasmic Ca^2+^ levels using the fluorescent calcium indicator fura-2 AM. Cells of the melanoma cell line Mel Im (derived from metastasis) were treated with 1 mM nitrate or 2 mM nitrite in a physiological HEPES-buffered solution. However, no alterations of cytoplasmic calcium concentrations were observed. Since previous studies revealed CAP-induced acidification to be essential for cellular effects of plasma-treated solutions, we combined acidic buffer solutions (pH 3.9) with nitrate and nitrite to resemble these aspects of CAP treatment. Interestingly, a synergistic effect of acidification and nitrite was observed, leading to a strong and immediate increase in cytoplasmic Ca^2+^ fluorescence (Figure 2A). Acidification alone, however, did not show significant effects. We also investigated the combination of nitrate and acidification but could not detect any increase in cytoplasmic calcium levels (Figure S3). Functional consequences of treatment with acidic nitrite solution were additionally analyzed using the melanoma cell line Mel Juso (derived from the primary tumor). Cell viability was assessed using the tetrazolium-based XTT assay, revealing significant cytotoxicity of a 5 min treatment in Mel Juso (Figure 2B) and Mel Im (Figure S4A). To exclude any contribution of HEPES to this effect, we repeated the experiment with a phosphate-buffered solution without HEPES. Similar cytotoxicity was observed, indicating that HEPES does not actively contribute to the effects of acidic nitrite solutions (Figure 2C and Figure S4B). Since phosphate-buffered solutions are not particularly suited for such low pH levels, all further experiments of this study used HEPES-buffered solutions. On a molecular level, cytotoxicity was accompanied by a strong induction of cell cycle inhibitor p21, which was found both on mRNA and protein levels (Figure 2D–F and Figure S4C–E). Immunofluorescent stainings of promyelocytic leukemia protein (PML) were used to evaluate DNA damage in Mel Juso. The number of PML nuclear bodies was significantly increased in response to treatment with acidic nitrite solution (Figure 2G,H). Finally, we assessed whether acidic nitrite solutions exhibited the same tumor selectivity that was previously reported for CAP. As expected, normal human fibroblasts showed no significant response to the treatment as compared to melanoma cell lines Mel Juso and Mel Im (Figure S4F).

### 2.3. Molecular Effects of Acidic Nitrite Solution Compared to CAP Treatment

**Figure 3:**
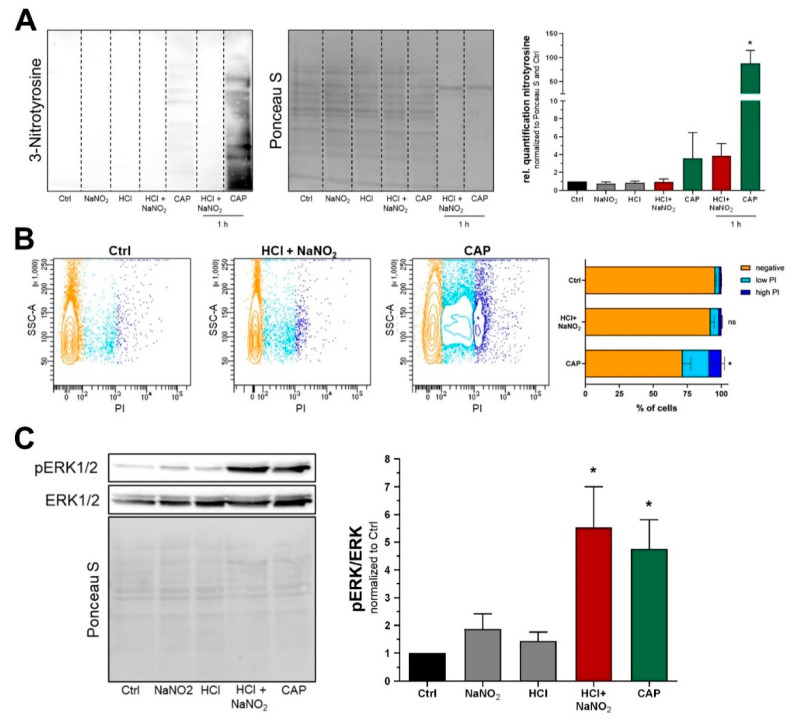
Molecular effects of acidic nitrite solution and CAP. (**A**) Western blot analysis of protein nitration directly after 5 min treatment with acidified nitrite solution or indirect CAP (F_(6,14)_ = 9.437, *p* = 0.0003). Reference samples of 1 h treatment serve as a positive control. (**B**) Propidium iodide staining in combination with flow cytometry to assess membrane damage after treatment (F_(2,6)_ = 11.62, *p* = 0.0086). (**C**) Western blot analysis of pERK1/2 and ERK1/2 after treatment with acidic nitrite solution or indirect CAP (F_(4,10)_ = 7.404, *p* = 0.0049). Control treatment (Ctrl) refers to ECS without nitrite at pH 7.4. Bars are shown as mean ± SEM (ANOVA followed by Tukey’s HSD post-hoc test vs. Ctrl, *n* = 3, *: *p* < 0.05).

Multiple studies proposed the generation of peroxynitrite (ONOO-) to be a major consequence of CAP treatment [30,31]. We aimed to estimate intracellular ONOO- generation indirectly via quantification of 3-nitrotyrosin, a marker that was previously reported to be a major consequence of peroxynitrite-dependent protein nitration [32,33]. Therefore, Western blot analysis of 3-nitrotyrosine was performed directly after a 5 min treatment with acidic nitrite solution (Figure 3A). Interestingly, we could not detect relevant amounts of 3-nitrotyrosine even after prolonged treatment periods of 1 h. A physiological buffer solution treated with 2 min CAP was used as a positive control and resulted in protein nitration after it was applied on the cells for 5 min and strong induction of 3-nitrotyrosine after 1 h incubation. Another established feature of CAP was the introduction of membrane damage, which was assessed next. We used a 5 min propidium iodide (PI) staining without fixation or permeabilization to detect ruptures in the cellular membrane. While there was a minor increase in PI signal after treatment with acidic nitrite solution, we could not find significant induction of membrane damage (Figure 3B). Again, a buffer solution treated with 2 min CAP was used as a positive control and led to a significant increase in PI signal. Next, we analyzed MAPK activity by Western blot analysis of ERK1/2 phosphorylation and found a significant elevation of pERK1/2 after 5 min treatment with acidic nitrite solution or indirect CAP treatment (Figure 3C).

## 3. Discussion

Generation of nitrite and nitrate after CAP treatment was addressed in some publications before, but their presence and quantity highly depend on the used plasma device and experimental conditions. Since we wanted to combine these molecules with the previously reported acidification after CAP treatment, detection and quantification of both molecules were an essential part of developing a valid RNS-based treatment comparable to CAP. We used Raman spectroscopy to identify long-lived reactive species and found increased levels of nitrate and nitrite, which were then quantified. The use of Raman spectroscopy has shown to be especially practicable due to the all-optical assessment allowing contact-free and label-free quantification of samples. Our results of up to mM ranges are supported by studies on plasma-treated aqueous solutions using other plasma devices [34,35] and display an important baseline for comparison of CAP effects.

When monitoring cytoplasmic calcium levels in melanoma cells, we detected a significant difference between treatments using acidic nitrite and nitrate solutions. It, therefore, seems important to differentiate between these two species when assessing RNS-related effects of CAP, which is not reliably done to date. While it is possible that nitrate contributes to the CAP effect on melanoma cells, most probably due to interaction with ROS and other components of the plasma [36], its role is most definitely minor in comparison to acidic nitrite solutions.

The combination of inorganic nitrite and acidification was previously referred to as *acidified nitrite* [37,38,39]. On a molecular level, such treatment mainly results in the generation of nitrous acid (HNO_2_), an unstable compound that degrades to NO and NO_2_. However, the strong acidification might result in further protonation and production of additional reactive species such as H_2_NO_2_^+^ or N_2_O_3_ [40,41]. Identification and evaluation of these reactive species were not addressed in this study, but since the CAP and nitrite effects both depend on strong extracellular acidification, it is possible that their cytotoxicity is not solely based on HNO_2_. Acidified nitrite solutions have been well studied due to their antimicrobial activity and positive effects on wound healing, including successful clinical trials using acidified nitrite creams [42,43]. The similarity of these effects with CAP indicates a potential role during plasma treatment. However, the antitumor effects of acidified nitrite are mostly unknown to date. To our knowledge, there is only one study by Morcos et al. [44] showing that 50 µM sodium nitrite inhibits human bladder tumor cells at pH 5.5 to 6 by interfering with DNA replication. However, due to the narrow methodological spectrum of this study and the focus on a physiological rather than therapeutic setting, their work was hardly proof of antitumor effects of acidified nitrite. Nevertheless, it supports our findings and indicates that such treatment might be able to inhibit a wide variety of cancer cells. In the present study, acidified nitrite caused a strong reduction of cell viability in human melanoma cells. Surviving cells were characterized by significant DNA damage and activation of cell cycle inhibitor p21, thereby indicating that this treatment causes lasting damage to melanoma cells. Due to these severe cytotoxic effects, we propose that acidification and nitrite are important components of CAP. Cytotoxicity in normal human fibroblasts was not significant, unlike both melanoma cell lines used in this study, indicating tumor selectivity similar to CAP. However, further comparative studies will be necessary to validate this observation. It was previously reported that HEPES might undergo chemical changes in response to reactive species [45,46]. Since the products of such reaction were found to be cytotoxic, it was necessary to rule out any contribution of modified HEPES to the effects observed in this study. The cytotoxicity of acidified nitrite, however, was still present after interchanging HEPES with a phosphate buffer. We, therefore, conclude that molecular changes of HEPES play a negligible role during our experiments. Thus far, molecular studies of CAP show a strong tendency towards ROS, mainly due to their superior reactivity and cytotoxicity in comparison to RNS. It is, therefore, not surprising that previous studies on CAP-induced nitrite only used it in combination with ROS (namely H_2_O_2_) to assess antitumor effects [47,48].

On a molecular level, CAP effects on tumor cells were previously linked to oxidative stress, such as the generation of peroxynitrite (ONOO^−^) and the resulting formation of nitrated proteins. For example, we recently reported increased levels of 3-nitrotyrosine after short CAP treatment of melanoma cells [28]. Furthermore, several studies proposed a causative role of ONOO^−^ during antimicrobial and even cytotoxic effects of CAP [49,50]. In the present work, we used an experimental setup solely based on RNS and could not detect significant amounts of protein nitration, most probably due to the absence of ROS. Nevertheless, acidified nitrite was found to be strongly cytotoxic, indicating that molecular mechanisms independent of peroxynitrite exist and might also be involved in CAP effects. Another common feature of CAP is the induction of membrane damage in prokaryotic and eukaryotic cells [51,52]. Such alterations of the plasma membrane were previously used to improve drug delivery to cells and through tissues [53] but also have a potential role in antimicrobial and antitumor effects. Interestingly, we could not detect increased membrane damage in response to treatment with acidified nitrite. A possible explanation can be found in the studies of He et al. [52,54], which proposed ROS-dependent lipid peroxidation as the main cause of CAP-induced membrane damage. Our findings of strong cytotoxicity without membrane damage suggest that this process might not be a major contributor to CAP-induced killing of cancer cells. At this stage, however, we are not able to draw a final conclusion on the importance of protein nitration and membrane damage during the antitumor effects of CAP. It is likely that CAP utilizes a broader range of molecular mechanisms to induce cell death, some of which may depend on protein nitration or membrane damage.

When assessing signaling pathways, we found phosphorylation of ERK1/2 to be increased in response to CAP treatment, which was not described before in tumor cells. However, a few articles reported such an activation of the MAPK pathway in normal cells, leading to diverse cellular effects [55,56,57]. Since a similar increase in pERK1/2 was found after treatment with acidified nitrite, the MAPK pathway might be involved in the observed antitumor effects. This hypothesis is supported by an already established linkage between MAPK activation and apoptosis [58,59].

In summary, this study highlights the importance of acidified nitrite during CAP treatment and calls for further research on CAP-induced RNS. We could show that acidified nitrite is a potent inhibitor of melanoma cells, although it represents only a fraction of all reactive species involved in CAP. Additionally, the comparison of acidified nitrite and CAP treatment is a useful approach for the identification of molecular mechanisms and their evaluation in the context of antitumor effects. Our observations, therefore, contribute to a better understanding of CAP action on tumor cells and facilitate development of CAP-based anti-cancer therapies.

## 4. Materials and Methods

### 4.1. Chemicals and Solutions

Extracellular solution (ECS) and phosphate-buffered ECS (pbECS) were prepared as previously described [28]. For ECS, the following chemicals were diluted in bi-distilled water: 145 mM NaCl, 5 mM KCl, 10 mM glucose, 1.25 mM CaCl_2_, 1 mM MgCl_2_, and 10 mM HEPES. For preparation of pbECS, 133 mM NaCl, 3.5 mM KCl, 10 mM glucose, 1.25 mM CaCl_2_, 1 mM MgCl_2_, 1.5 mM KH_2_PO_4_, and 8.1 mM Na_2_HPO_4_ were diluted in bi-distilled water. Both solutions were adjusted to pH 7.4. Sources for further chemicals: Fura-2 AM and pluronic F-127 (Biotium, Fremont, CA, USA), ionomycin (Enzo Life Sciences, Farmingdale, NY, USA), KNO_3_ (Carl Roth, Karlsruhe, Germany), NaNO_3_ (Acros Organics, Fair Lawn, NJ, USA), NaNO_2_ (Sigma Aldrich, Steinheim, Germany), salicylic acid (Carl Roth), Sulfuric acid (Carl Roth, Karlsruhe, Germany), sulfanilamide (Sigma Aldrich, Steinheim, Germany), naphthylethylenediamine dihydrochloride (Sigma Aldrich, Steinheim, Germany).

### 4.2. CAP Treatment

CAP treatment of aqueous solutions was described previously [28]. Eight droplets of ECS (20 µL each) were distributed evenly inside a 35 mm petri dish. A MiniFlatPlaster device was placed directly above the dish, resulting in a distance of approximately 10 mm between electrode and sample. Air circulation was minimized by contact of the device to the plastic dish with careful application of pressure. After treatment was finished, all droplets were collected and transferred to reaction tubes for further processing. ECS treated with 2 min CAP was used for the indirect treatment of melanoma cells.

### 4.3. Raman Spectroscopy

A laser diode with a wavelength of 785 nm (Laser-785-LAB-ADJ-S, Ocean Optics, Dunedin, FL, USA) was used in combination with a low noise spectrometer (QE65000 Pro-Raman, Ocean Optics, Dunedin, FL, USA) and a Raman probe (General Purpose Raman, RIP-RPB-785-SMA-SMA, Ocean Optics, Dunedin, FL, USA). Before each measurement, a reference ‘dark spectrum’ was recorded and subtracted. An enclosed aluminum chamber was used to measure liquid samples (Figure S5). In this custom design, a 100 µL sample carrier was placed at the working distance of the Raman probe. The molecular concentration of each solution was quantified before the experiment, and a negative control sample of ultra-pure water (Merck-Millipore Chemicals GmbH, Darmstadt, Germany) served as negative control (0 mmol). From each solution, 100 subsequent spectra were recorded with 2 s integration time each. Data processing of the Raman spectra was performed as previously described [60]. The raw spectra were cropped to the spectral range 500–1500 cm^−1^. Denoising was achieved by a median filter and discrete wavelet denoising (DWT) (k = 2, J_max_ = 2) [61]. The autofluorescence background was modeled by asymmetric least square [62] with λ = 73 and *p* = 0.001 and then subtracted. Finally, each spectrum was normalized to its entire area under the curve. The peak value was determined within the spectral resolution of 12 cm^−1^.

### 4.4. Photometric Nitrate Assay

Quantification of nitrate was based on the transnitration of salicylic acid. A volume of 2 µL of the sample solution was combined with 8 µL of 5% salicylic acid in concentrated sulfuric acid and allowed to incubate for 20 min. Then, 200 µL of 8% NaOH was added to achieve basic pH. The solution was mixed thoroughly, measured at 410 nm using a Clariostar Plus Multiplate reader (BMG Labtech, Ortenberg, Germany), and quantified using a standard curve of NaNO_3_.

### 4.5. Photometric Nitrite Assay

A modified Griess diazotization reaction was used to quantify nitrite levels. Briefly, 1.5 µL of the sample solution was transferred to a 96-well plate, followed by 100 µL 1% sulfanilamide in 1 M HCl and 100 µL 0.2% naphthylethylenediamine dihydrochloride (NED) in bi-distilled water. After 15 min incubation, the solution was mixed thoroughly, measured at 540 nm using a Clariostar Plus Multiplate reader, and quantified using a standard curve of NaNO_2_.

### 4.6. Cell Culture

Melanoma cell line Mel Im and normal human fibroblasts were cultivated in DMEM low glucose, while the Mel Juso cell line required RPMI 1640 medium with 2% sodium bicarbonate. All media were supplemented with 10% FCS and 1% penicillin/streptomycin. Cells were incubated at 37 °C and 8% CO_2_ until approximately 80% confluence. Following a washing step with PBS, a solution of 0.05% trypsin and 0.02% EDTA in PBS was applied to detach the cells. After centrifugation and removal of the trypsin solution, cells were counted using a Neubauer counting chamber. Mycoplasma contamination was regularly excluded for all cell lines. All cell culture chemicals and media were obtained from Sigma Aldrich.

### 4.7. Calcium Imaging

The experimental setup and procedures were described elsewhere [27]. Briefly, 200,000 cells were seeded in 35 mm cell culture dishes. On the next day, cells were stained with fura-2 AM (3 µM) in ECS with 0.02% pluronic for 30 min at 37 °C and 8% CO_2_. After a 5 min washing step with ECS, the solution was removed before the dish was mounted on an inverted microscope, and the perfusion outlet was placed within 1 mm distance to the cells. The imaging procedure started with 1 min background measurement, followed by 4 min treatment with the sample solution. During this time, cells were alternatingly excited at 358 nm and 391 nm while recording fura-2 fluorescence. Intracellular calcium levels were evaluated by calculation of the F358 nm/F391 nm ratio. The area under the curve (AUC) refers to the first 120 s after treatment began, relative to the fluorescence 10 s before treatment. To ensure responsiveness of the cells and validate the staining, 2 µM ionomycin was applied after the treatment. Consequently, non-responsive or erratic cells were excluded from analysis. Areas of interest were placed on individual cells, and their fluorescence ratio time courses were calculated after background subtraction. Further information on data evaluation and imaging equipment can be found in a previous publication [63].

### 4.8. Nitrite Treatment

A stock solution of 50 mM NaNO_2_ was prepared freshly using ECS or pbECS with the according pH. Cells were washed with PBS to remove all cell culture media and cell debris, followed by the addition of a 2 mM NaNO_2_ solution. Unless otherwise specified, treatment duration was 5 min at 37 °C. The solution was removed afterward, and cells were cultivated for 24–72 h in their regular cell culture medium.

### 4.9. Cell Viability Assay

One day prior to treatment, 6000 cells/well were seeded in a 96-well plate. Following treatment and subsequent incubation for 24 h, cell viability was assessed using the Cell Proliferation Kit II (Roche, Basel, Switzerland) according to the manufacturer’s instructions. Photometric detection was realized with a Clariostar Plus Multiplate reader. Absorbance values were normalized to control. The resulting ratios were visualized as % of control.

### 4.10. Analysis of mRNA Expression by Real-Time PCR

Approximately 150,000 cells/well were seeded in 6-well plates 1 day before treatment. RNA isolation was performed 24 h, 48 h, and 72 h after treatment using E.Z.N.A.^®^ Total RNA Kit (Omega Bio-Tek, Norcross, GA, USA) according to manufacturer’s instructions, followed by cDNA generation using reverse transcriptase reaction as previously described [64]. Real-time PCR was carried out in LightCycler^®^ 480 II devices (Roche, Basel, Switzerland) with forward and reverse primers from Sigma-Aldrich: p21_for: 5′-CGAGGCACCGAGGCACTCAGAGG-3′; p21_rev: 5′-CCTGCCTCCTCCCAACTCATCCC-3′; 18s_for: 5′-TCTGTGATGCCCTTAGATGTCC-3′; 18s_rev: 5′-CCATCCAATCGGTAGTAGCG-3′.

### 4.11. Western Blot Protein Analysis

Approximately 150,000 cells/well were seeded in 6-well plates 1 day before treatment. Total protein isolation was performed 24 h, 48 h, and 72 h after treatment by addition of radio-immunoprecipitation assay buffer (Roche, Basel, Switzerland) as described elsewhere [65]. Detection of 3-nitrotyrosin required immediate protein isolation after treatment. 20 µg protein were loaded on a 10.00% or 12.75% SDS polyacrylamide gel for electrophoresis and subsequently blotted onto a PVDF membrane (Bio-Rad, Hercules, CA, USA). After a short incubation in methanol, Ponceau S staining was performed to quantify total protein load. Membranes were then washed with double distilled water and incubated in 5% non-fat dried milk/TBS-T for 1 h to block unspecific binding sites. Primary antibodies against p21 (1:5000 in 5% NFDM, Abcam, ab109199), 3-nitrotyrosine (1:1000 in TBST, Merck Millipore, 06-284), pERK and ERK (1:1000 in 5% BSA, Cell Signaling, 4370 and 9102) were incubated overnight shaking at 4 °C. Secondary antibodies conjugated to horseradish peroxidase (HRP, Cell Signaling, 7074) were applied for 1 h at room temperature. Visualization of HRP-conjugated antibodies was achieved by the addition of Clarity™ Western ECL Substrate (Bio-Rad) in combination with a Chemostar chemiluminescence imager (Intas, Goettingen, Germany). Signal intensity was then quantified using LabImage software version 4.2.3 (Kapelan Bio-Imaging GmbH, Leipzig, Germany).

### 4.12. Immunofluorescent Staining

Approximately 35,000 cells were seeded on 18 mm round coverslips the day before treatment. 24 h after treatment, cells were fixed and stained as previously described [66]. The following antibodies were used: anti-PML (1:200, Santa Cruz, Dallas, TX, USA), Cy3 anti-mouse (1:400, Thermo Fisher, Waltham, MA, USA). Cells were eventually stained with DAPI (1:10,000, Sigma Aldrich, Steinheim, Germany). Final stainings were stored at 4 °C and analyzed using an Olympus IX83 inverted microscope in combination with Olympus CellSens Dimension software (Olympus, Tokio, Japan).

### 4.13. Detection of Membrane Damage

Approximately 200,000 cells/well were seeded in 6-well plates. After cultivation for 24 h, cells were washed with PBS, treated with each sample solution, and washed again. Staining was achieved by the addition of 1 mL propidium iodide solution (10 µg/mL, PromoCell, Heidelberg, Germany) and 5 min incubation at room temperature. The staining solution was then removed, cells were washed with PBS and detached from the plate using trypsin. Following another washing step, cells were eventually resuspended in 1% BSA/PBS and analyzed by flow cytometry (LSRFortessa^TM^, BD Biosciences, San Jose, CA, USA). Data analysis was done using FACSDiva 9.0 software (BD Biosciences).

### 4.14. Statistical Analysis

Experimental results were analyzed and visualized using GraphPad Prism 7 software (GraphPad Software Inc., San Diego, CA, USA). If not otherwise specified, at least 3 biological replicates were measured, and statistical analysis was performed by one-way ANOVA. A significant F-test was followed by Tukey’s HSD post-hoc tests. A critical value of *p* < 0.05 was set for statistical significance. All results were given as mean ± standard error of the mean (SEM).

## Data Availability

Not applicable.

## Supplementary Materials

The following are available online at https://www.mdpi.com/article/10.3390/ijms22073757/s1, Figure S1: Calibration of Raman spectroscopy to potassium nitrate solutions of known concentration, Figure S2: Calibration of Raman spectroscopy to sodium nitrite solutions of known concentration, Figure S3: Acidified nitrate solution does not induce a cytoplasmic Ca^2+^ release in melanoma cells, Figure S4: Effects of acidic nitrite solution in melanoma cell line Mel Im and normal human fibroblasts, Figure S5: Custom designed experimental chamber for Raman spectroscopy.
