# Supplementary material for: Acidified Nitrite Contributes to the Antitumor Effect of Cold Atmospheric Plasma on Melanoma Cells
Source: Int J Mol Sci. 2021 Apr 4;22(7):3757. doi: 10.3390/ijms22073757 (PMC8038463; doi:10.3390/ijms22073757)
Supplement: Supplementary file 1 [file ijms-22-03757-s001.pdf]

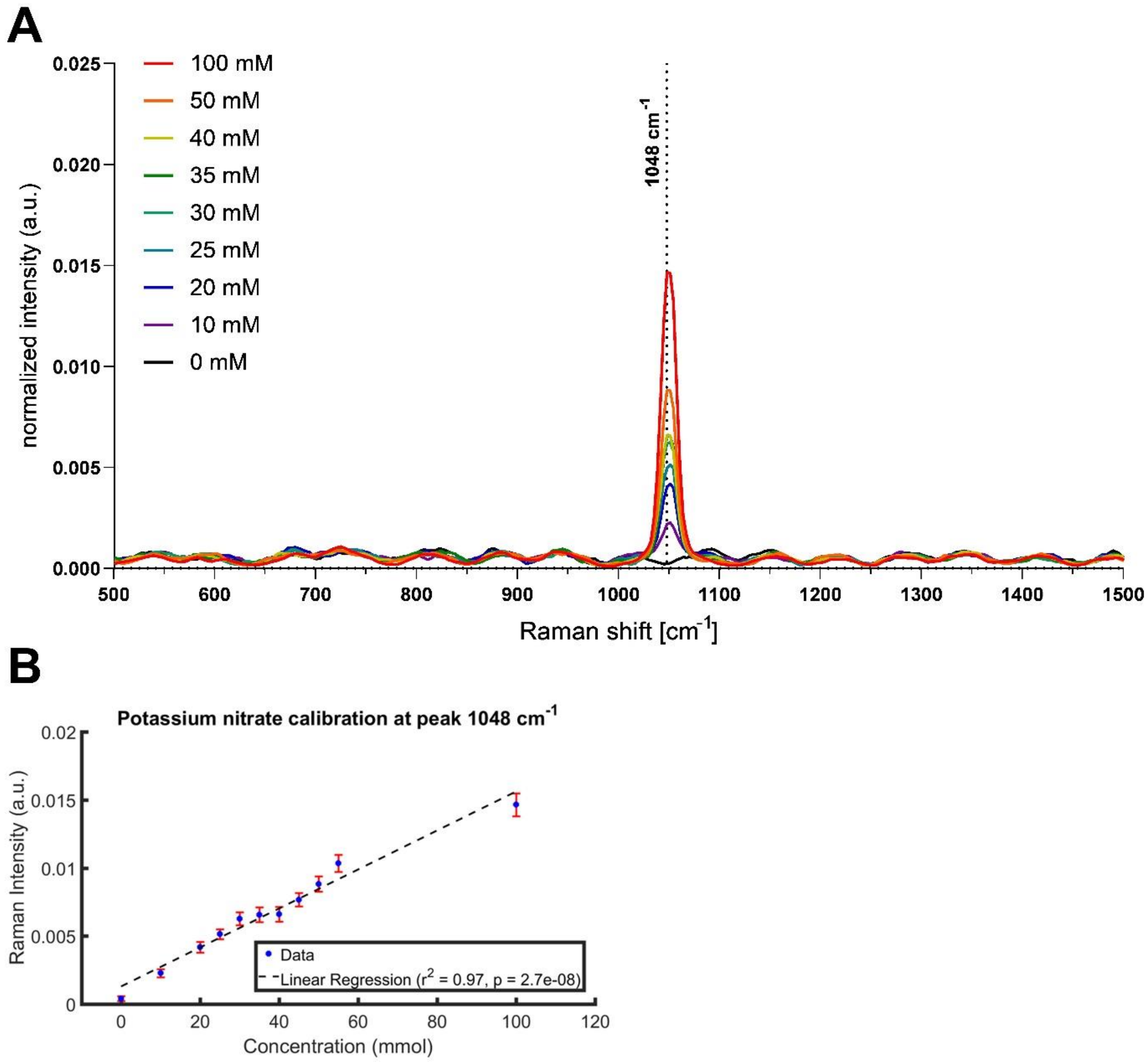

Figure S1. Calibration of Raman spectroscopy to potassium nitrate solutions of known concentration. (**A**) Average Raman spectra of the different concentrations. (**B**) Raman intensity of these solutions at 1048 cm^{-1}. Data are shown as mean with standard deviation error bars (n=100). A linear regression model is shown as dotted line and Anova with subsequent F-test were performed to determine r^2 and p value.

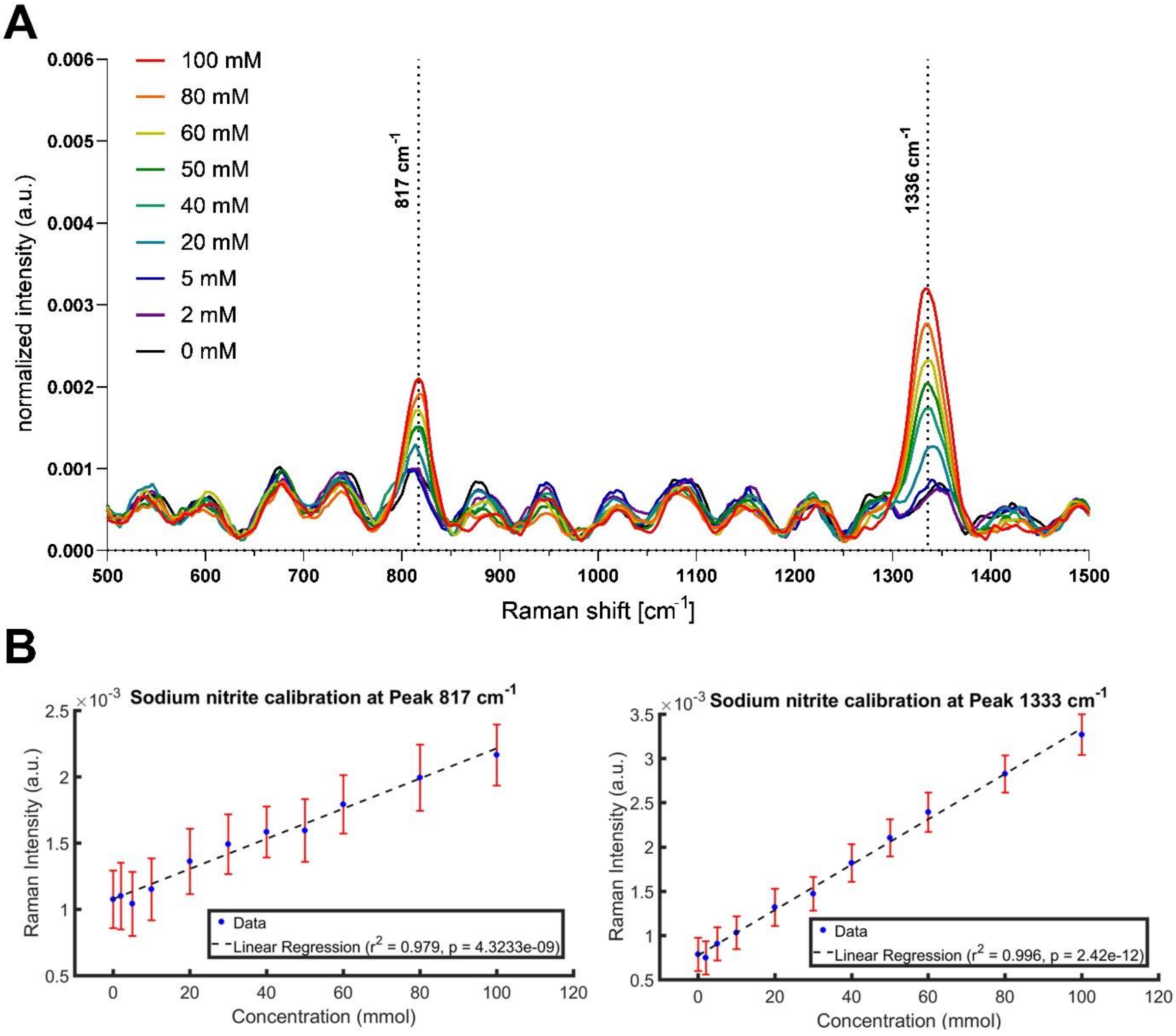

Figure S2. Calibration of Raman spectroscopy to sodium nitrite solutions of known concentration. (**A**) Average Raman spectra of the different concentrations. (**B**) Raman intensity of these solutions at 817 cm^{-1} and 1336 cm^{-1}. Data are shown as mean with standard deviation error bars (n=100). A linear regression model is shown as dotted line and Anova with subsequent F-test were performed to determine r^2 and p value.

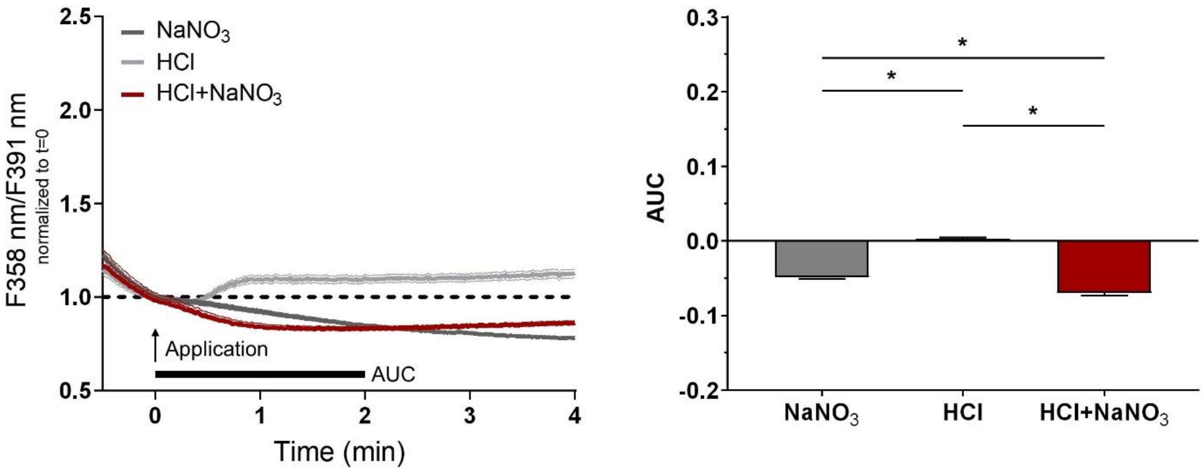

Figure S3. Acidic nitrate solution does not induce a cytoplasmic Ca^{2+} release in melanoma cells. Time course of cytoplasmic Ca^{2+} levels due to a 4-minute treatment with acidic ECS (HCl), nitrate solution ($NaNO_3$), or a combination of both. Traces are mean with 95% confidence interval, bars are mean ± SEM (ANOVA followed by Tukey's HSD post-hoc test, $F_{(2,863)} = 242.4$, $p < 0,0001$, n= 286 – 293, *: $p < 0.05$).

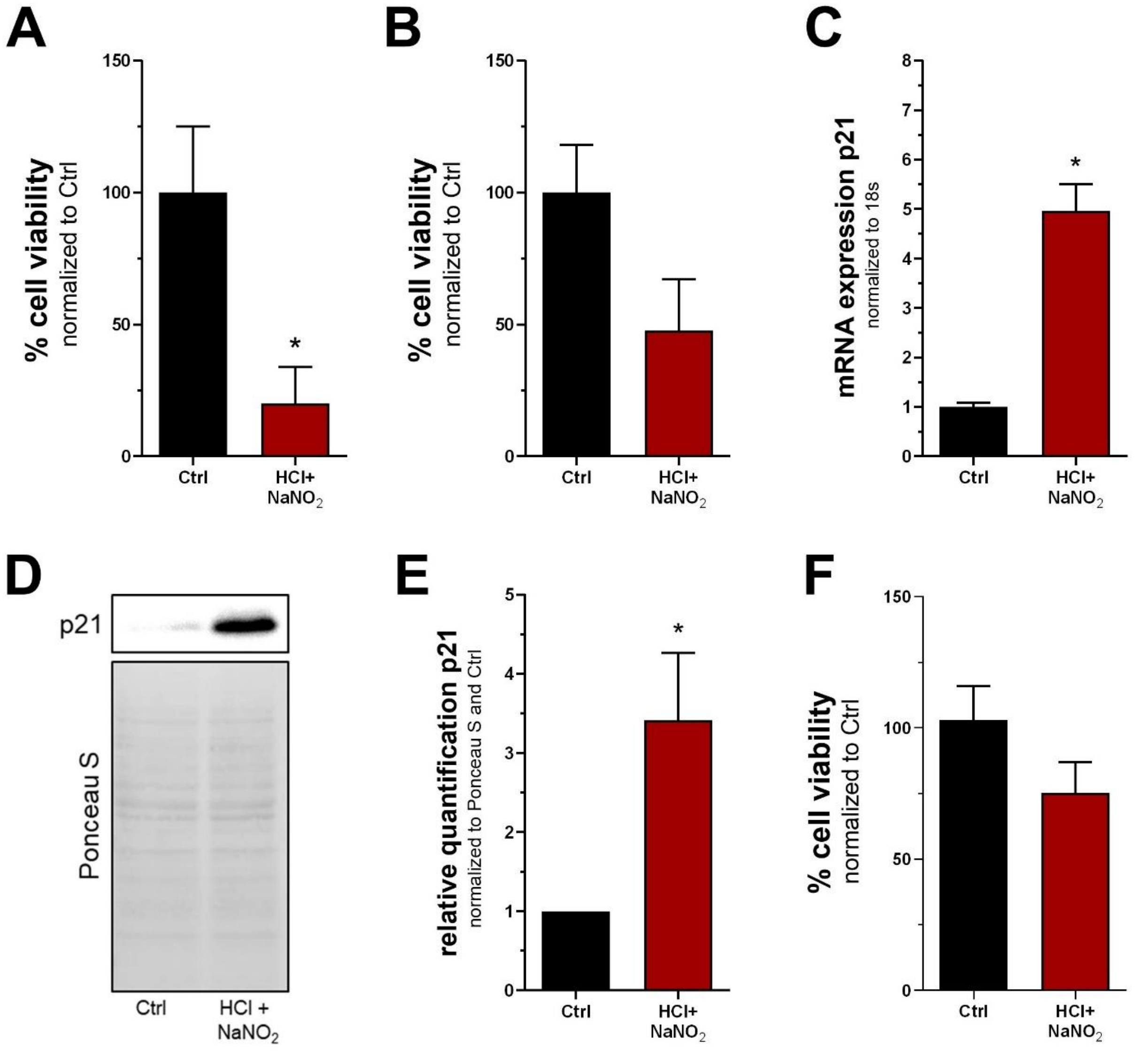

Figure S4. Effects of acidic nitrite solution in melanoma cell line Mel Im (**A-E**) and normal human fibroblasts (**F**). (**A**) Cell viability analysis 24 h after a 5 min treatment with physiological ECS (Ctrl) or a combination of acidic ECS and nitrite. (**B**) Cell viability as described in (**A**), but with HEPES-free pbECS. Every other experiment in this figure was done using HEPES-buffered ECS. Expression (**C**) as well as Western blot **(D,E**) analysis of p21 24 h after 5 min treatment with acidic nitrite solution. (**F**) Cell viability analysis of normal human fibroblasts 24 h after 5 min treatment with physiological ECS or a combination of acidic ECS and nitrite. Bars are mean ± SEM (Student's t-test, n=3, *: $p < 0.05$).

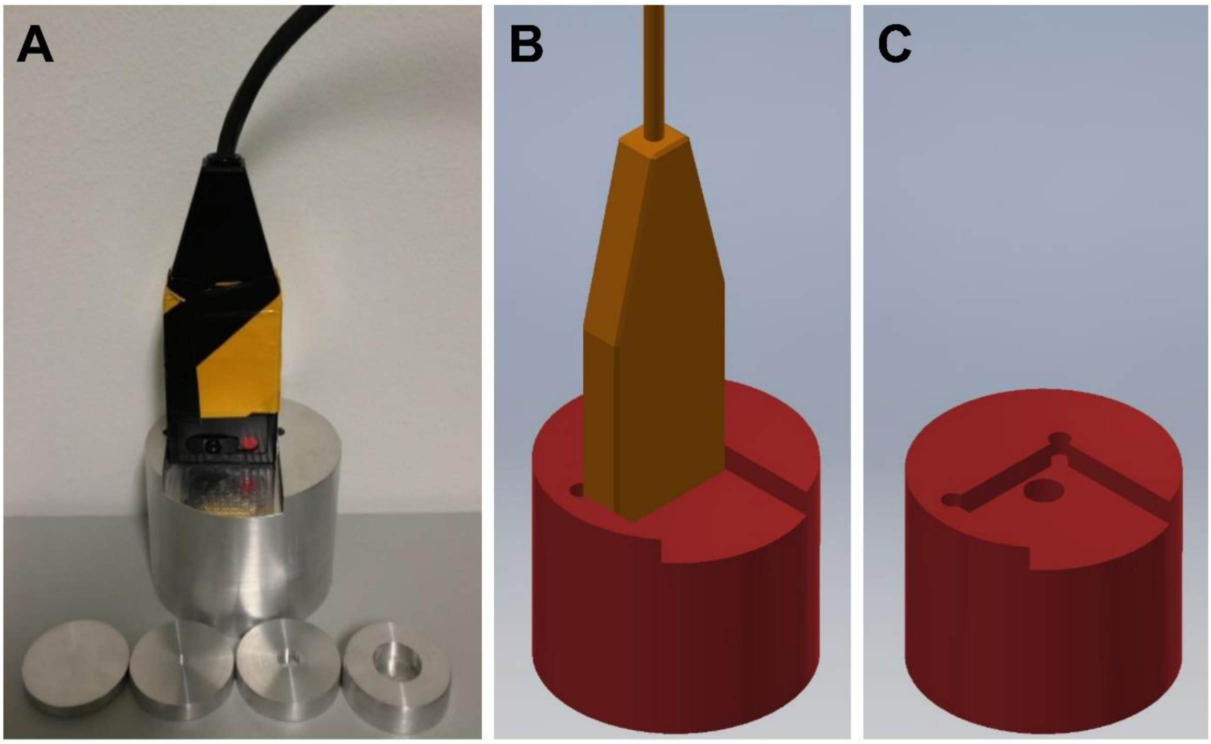

Figure S5. Custom designed experimental chamber for Raman spectroscopy. (**A**) The Raman probe inside the aluminum chamber with several carriers of different sample volume. (**B,C**) CAD model of the chamber with and without the probe.
